# Association between sleep disturbances and suicidal behavior in adolescents: a systematic review and meta-analysis

**DOI:** 10.3389/fpsyt.2024.1341686

**Published:** 2024-10-02

**Authors:** Valentina Baldini, Martina Gnazzo, Giada Rapelli, Mattia Marchi, Luca Pingani, Silvia Ferrari, Diana De Ronchi, Giorgia Varallo, Fabrizio Starace, Christian Franceschini, Alessandro Musetti, Michele Poletti, Giovanni Ostuzzi, Fabio Pizza, Gian Maria Galeazzi, Giuseppe Plazzi

**Affiliations:** ^1^ Department of Biomedical and Neuromotor Sciences, University of Bologna, Bologna, Italy; ^2^ Department of Biomedical, Metabolic and Neural Sciences, University of Modena and Reggio Emilia, Modena and Reggio Emilia, Italy; ^3^ Department of Psychology, University of Bologna, Bologna, Italy; ^4^ Dipartimento ad Attività Integrate di Salute Mentale e Dipendenze Patologiche, Azienda Unità Sanitaria Locale (USL) IRCCS Reggio Emilia, Reggio Emilia, Italy; ^5^ Department of Mental Health and Dependence, AUSL of Modena, Modena, Italy; ^6^ Department of Medicine and Surgery, University of Parma, Parma, Italy; ^7^ Department of Humanities, Social Sciences and Cultural Industries, University of Parma, Parma, Italy; ^8^ Department of Mental Health and Pathological Addiction, Child and Adolescent Neuropsychiatry Service, Azienda Unità Sanitaria Locale (USL)-IRCCS di Reggio Emilia, Reggio Emilia, Italy; ^9^ World Health Organiization (WHO) Collaborating Centre for Research and Training in Mental Health and Service Evaluation, Department of Neuroscience, Biomedicine and Movement Sciences, Section of Psychiatry, Verona, Italy; ^10^ IRCCS Istituto Delle Scienze Neurologiche di Bologna, Bologna, Italy

**Keywords:** suicide, sleep disturbances, adolescence, insomnia, sleep disorders, suicidal ideation

## Abstract

**Introduction:**

Adolescents’ health and well-being are seriously threatened by suicidal behaviors, which have become a severe social issue worldwide. Suicide is one of the leading causes of mortality for adolescents in low and middle-income countries, with approximately 67,000 teenagers committing suicide yearly. Although an association between sleep disturbances (SDs) and suicidal behaviors has been suggested, data are still scattered and inconclusive. Therefore, to further investigate this association, we conducted a meta-analysis to verify if there is a link between SDs and suicidal behaviors in adolescents without diagnosed psychiatric disorders.

**Methods:**

PubMed, CENTRAL, EMBASE, and PsycINFO were searched from inception to August 30th, 2024. We included studies reporting the estimation of suicidal behaviors in adolescents from 12 to 21 years of age, with SDs and healthy controls. The meta-analysis was based on odds ratio (OR, with a 95% confidence interval ([CI]), estimates through inverse variance models with random-effects.

**Results:**

The final selection consisted of 19 eligible studies from 9 countries, corresponding to 628,525 adolescents with SDs and 567,746 controls. We found that adolescents with SDs are more likely to attempt suicide (OR: 3.10; [95% CI: 2.43; 3.95]) and experience suicidal ideation (OR: 2.28; [95% CI 1.76; 2.94]) than controls.

**Conclusion:**

This meta-analysis suggests that SDs are an important risk factor for suicidal ideation and suicide attempts in healthy adolescents. The findings highlight the importance of early identification of SDs to prevent suicidal behaviors in this population.

**Systematic review registration:**

PROSPERO, identifier CRD42023415526.

## 1 Introduction

Suicide is estimated as the fourth cause of death for people between the ages of 13 and 29 (1). Suicidality entails a wide range of phenomena, from death wishes and thoughts, also known as suicidal ideation (SI), to actual suicidal behaviors, comprising suicide attempts (SA) and non-suicidal self-injury (NSSI), which refers to the intentional harm to the body with no death desire and completed suicide. SI is relatively common in adolescence, with nearly 20% of 12-year-olds reporting such feelings in the past month (2). Also, NSSI typically occurs during adolescence, with a prevalence of 17% compared to 5% in adulthood (3). Despite these statistics, the prevention of adolescent suicide has received limited attention compared with suicide prevention in adults.

Previous research has shown that self-injures not only repeatedly inflicts painful injures but, also affects their cognitive and neurodevelopment trajectories (4).

Suicide imposes significant socioeconomic burdens on families, communities, and nations (5). Risk factors associated with suicidal behaviors in adolescents are multifactorial, complex, and interrelated (6).

Sleep disturbances (SDs) have recently gained attention as important risk factors for suicidal behaviors. SDs are defined as subjective experiences of difficulty falling asleep, frequent awakenings, short sleep duration, restless sleep, nightmares, and anxiety dreams, which affect approximately 7.8% to 23.8% of adolescents (5–7). Bedtimes get later with each passing year during adolescence, partially due to biological factors, such as adjustments to the homeostatic sleep regulating system that give greater tolerance for sleep deprivation, and sociocultural factors as the newly acquired autonomy (8). The average sleep duration during the weekend for the youngest adolescents is about 8.4 hours and about 6.9 hours for the high school seniors (9). Adolescents’ hours of sleep are significantly less than those recommended by the National Sleep Foundation (10). Furthermore, an Italian study revealed a different sleep profile across age groups: 16-years-olds subjects showed the highest percentage of insufficient sleep and frequent nocturnal awakenings, those between 18 and 19 years had the highest rate of insufficient sleep and difficulty falling asleep, and adolescents 17-year-old presented an elevated difficulty in waking up in the morning (11).

In adolescents, impaired sleep is associated with various psychosocial issues, such as an increased risk for depressed mood and anxiety disorders (12). Possibly, these disorders result from impaired emotion regulation that follows SDs. Indeed, a recent study showed that impaired emotion regulation strategies, such as decreased problem-solving and rumination, mediated the relation between SDs and anxiety and mood disorders (13). Lack of sleep is also associated with several health risk behaviors, such as increased substance use, excessive use of electronic media, and physical inactivity (14).

Previous reviews and meta-analyses found an association between SDs and suicide, including SI, SA, and completed suicide among adults (15–19). This association is, understandably, significantly reported among adult individuals with psychiatric diagnoses, in particular, the comorbidity of SDs (i.e., insomnia, parasomnia, and sleep-related breathing disorders, but not hypersomnia) and mental disorders (i.e., schizophrenia, depression, panic disorder, and post-traumatic stress disorder) was associated to an increased risk of completed suicide, a twofold risk of presenting SI and a fourfold risk of SA (20). Sleep is hypothesized to impact SI due to neurobiological factors such as the impact of sleep on serotonin and other factors involved in mood regulation, as well as the impact of nightmares (21). Adolescence witnesses notable alterations in sleep physiology, including reduced slow-wave sleep and delayed sleep phase syndrome. These changes have been linked to the emergence of SDs, such as insomnia symptoms. However, the precise mechanisms through which these physiological changes influence suicidal behaviors require further investigation (22). Sleep deprivation, a common consequence of SDs, can impair frontal lobe cognitive function. This impairment leads to compromised judgment and impulse control, potentially increasing the likelihood of engaging in suicidal behaviors (23). Adolescents, with their still-developing frontal lobe and emotional regulation circuitry, may be particularly vulnerable to these effects. Consequently, teenage sleep loss may promote young suicidality by increased impulsivity linked to the frontal lobe and emotional regulation circuitry that isn’t fully developed (24). Evidence suggests a link between SDs and weakened serotonergic systems. Individuals who have attempted suicide exhibit a more pronounced decline in serotonin production in the prefrontal cortex. This neurotransmitter imbalance may contribute to suicidal tendencies (25). Sleep-deprived adolescents may exhibit heightened reactivity in the mesolimbic network when exposed to pleasure-inducing stimuli. This heightened reactivity could potentially increase the inclination toward engaging in health-risk behaviors (26).

Adolescence is often characterized by changes in sleep patterns, in particular, adolescents reported poor sleep quality also due to physiological changes in sleep-wake architecture that become particularly pronounced from the onset to the end of puberty (27, 28). A significant gender gap must be highlighted, as female individuals report worse sleep quality than males due to hormonal changes; adolescents with SDs report more depression, anxiety, anger, inattention, drug and alcohol use, and reduced school performance. Adolescents also report more tiredness, less energy, a worse perception of health, and symptoms such as headache, stomachache, and back pain (29–31). SDs may increase the risk of suicide through psychosomatic disorders, which already represent a low level of health and possible risk factors for suicide.

The conclusions cannot be applied directly to the adolescent population because of the specific substantial differences in sleep–wake patterns that are considerably different from those of adults.

Specifically, during adolescence, the circadian rhythm becomes delayed, and the homeostatic sleep pressure is reduced, which leads to a change in sleep-wake patterns with later onset of sleep. Beyond this biological modification, behavioral and social factors further contribute to later bedtimes, as well as the increased use of electronic media and less parental involvement in setting bedtimes (32).

Furthermore, among healthy adolescents, the association is still inconsistent. A study showed that increased sleep duration among adolescents was associated with a low probability of having a suicide plan (33). Liu and colleagues showed in their review that in cross-sectional analyses, adolescents with SDs were at higher risk of SI and SA than those without SDs, while prospective reports indicated that SDs in adolescents significantly predicted the risk of SI but not of SA; finally, the retrospective study did not support the association between SDs and SA (34).

Therefore, considering that the association between SDs and suicidal behaviors could impact future suicide prevention strategies in adolescents and given the literature gaps, this meta-analysis aims to comprehensively examine the literature on the association between SDs and suicidal behaviors among adolescents without a formal psychiatric diagnosis.

## 2 Methods

The protocol of this study was registered in PROSPERO (CRD42023415526) and followed the Preferred Reporting Items for Systematics Review and Meta-analyses (PRISMA) reporting guidelines (35).

### 2.1 Eligibility criteria and search strategy

A literature search was carried out through the electronic databases PubMed, EMBASE, CENTRAL, and PsycINFO from inception to August 30th, 2024 (search terms are tailed in the **Supplementary Material**). The literature search was restricted to the English language and peer-reviewed journals.

Studies were included if they reported data on suicidal behavior, including SA, SI, NSSI, and death by suicide in individuals exposed to SDs compared to individuals unexposed. SDs were identified based on whether insomnia symptoms or other sleep problems were evaluated. Specifically, SDs were examined through specific questionnaires if they were available or through the hours of sleep.

We included studies on participants aged between 12 and 21 years without limitation of gender or ethnicity. To focus on the effect of SDs on suicidal behaviors and to minimize the confounding impact of concurrent psychiatric disorders, we excluded the studies involving participants with any formal psychiatric conditions. In particular, we did not include adolescents with a previous diagnosis of Major Depressive Disorders, Anxiety Disorder, Schizophrenia, or other mental illnesses certified by a psychiatrist.

A couple of authors (among VB, MG, and GR) independently screened titles, abstracts, and full text. A third author (MM) resolved the discrepancies by discussion and adjudication.

### 2.2 Data extraction and study quality assessment

A meta-analysis of the overall comparison of suicidal behavior rates among people with and without SDs was performed. Pooled Odds Ratios (ORs) with 95% confidence intervals (95% CIs) were generated using inverse variance models with random effects (36). The results were summarized using forest plots. Standard Q tests and the I^2^ statistic (i.e., the percentage of variability in prevalence estimates attributable to heterogeneity rather than sampling error or chance, with values of I^2^ ≥75% indicating high heterogeneity) were used to assess between-study heterogeneity (37). Leave-one-out analysis and meta-regression were performed to examine sources of between-study heterogeneity on a range of study-prespecified characteristics (i.e., sex, age, risk of bias, use of alcohol or drugs, cigarette smoking, school attainment, and bullying experience).

If the meta-analysis included more than ten studies, we performed funnel plot analysis and the Egger test to test for publication bias (38). The Egger test quantifies bias captured in the funnel plot analysis using the value of effect sizes and their precision (i.e., the standard errors [SE]) and assumes that the quality of study conduct is independent of study size. If analyses showed a significant risk of publication bias, we would use the trim and fill method to estimate the number of missing studies and the adjusted effect size (39–42). All the analyses were performed in R (RStudio 2021) using *meta* and *metafor* packages (43, 44). Statistical tests were 2-sided and used a significance threshold of p-value <0.05.

The studies included in the final section were assessed with the Newcastle-Ottawa Scale (NOS), which calculates the risk of bias in observational studies on the three domains (selection, comparability, and exposure) and provides an overall score ranging from 1 (the highest risk of bias) to 9 (the lowest risk of bias). Two authors (MG, GR) assessed the risk independently, and disagreements were discussed with a third author (VB).

## 3 Results

### 3.1 *Characteristics* of included studies

**Figure 1:**
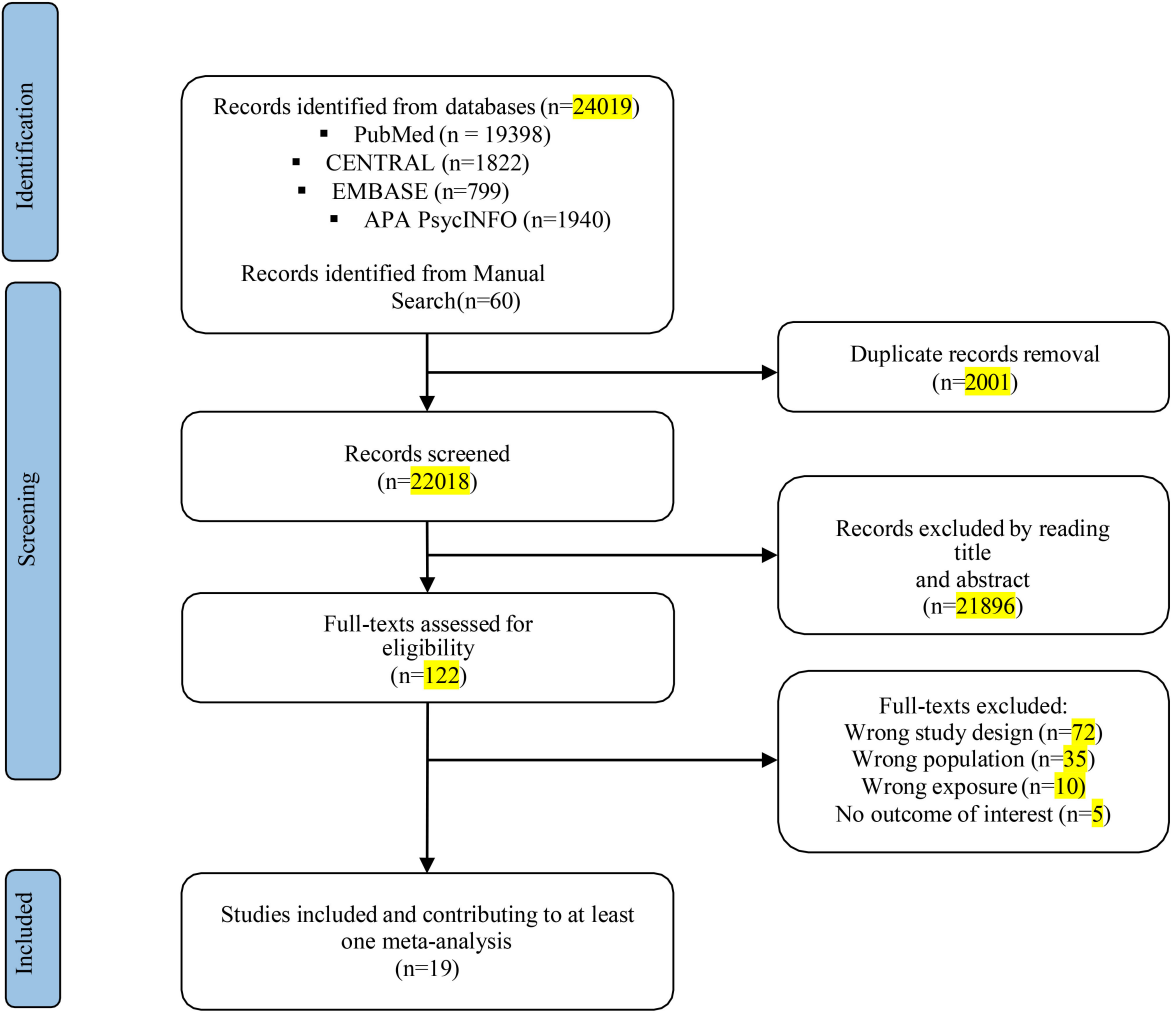
Flow-chart describing the study selection process.

The literature search using electronic methods resulted in a total of 24,019 records. After removing duplicate entries, 22,018 records were subjected to title and abstract screening. After the preliminary stage, 122 completed texts were evaluated for eligibility. Of these, 19 studies met the pre-specified inclusion criteria. These studies encompassed a sample size of 628,525 adolescents with SDs (including insomnia symptoms or other sleep problems) and 567,746 control participants (see **Figure 1**). **Table 1** presents a comprehensive overview of the participant characteristics observed in the studies included in the analysis. The studies included in this analysis quantify SDs in terms of hours and assess sleep quality. The characteristics of the studies were reported in **Table 2**.

**Table 1:** Study participants’ characteristics of included studies.

First author, year	Country	Mean age	Women (%)	Overall sample	N. events (any suicidal behavior)	Study design	Type of suicidal behavior	NOS overall score
Altangerel, 2014 (64)	Mongolia	12.5	NR	15.510	2.116	Cross-sectional	SA, SI	4
Baiden, 2020 (65)	USA	16	51.8	13.659	2.409	Cohort	SI	8
Chung, 2014 (66)	China	13	49.7	607	258	Cross- Sectional	SI	9
Dema, 2019 (80)	Bhutan	15	53.5	5.809	1.323	Cross-sectional	SA, SI	8
Fitzgerald, 2011 (67)	USA	16.2	47.9	12.154	3.974	Cross-sectional	SA, SI	8
Franic, 2014 (68)	Croatia	12.2	49.9	840	433	Cross-sectional	SI	9
Gong, 2020 (69)	China	12.2	49.2	1.513	308	Cross-sectional	SA, SI	9
Jacob, 2020 (70)	UK	15.1	52.1	9.352	4.676	Case-control	SA	6
Kim, 2015 (71)	Korea	15	48.2	191.642	52.665	Cross-sectional	SA, SI	4
Kim, 2021 (72)	Korea	15	49	475.575	19.285	Cross-sectional	SI	7
Lin, 2018 (73)	China	15	50.9	479.967	1.395	Cohort	SA	8
Liu, 2019 (20)	China	14.6	48.1	7.072	750	Cohort	SA	9
Liu, 2021 (36)	USA	15	49.9	6.923	839	Cohort	SA	9
Roane, 2008 (74)	USA	15.8	52.4	4.494	NR	Cross-sectional	SA, SI	8
Shi, 2021 (75)	China	20.1	49.9	11.740	1.385	Cross-sectional	SA, SI	9
Verkooijen, 2018 (76)	Germany	14.5	49.5	16.781	1.910	Cohort	SA, SI	6
Weis, 2015 (77)	Israel	21.6	74	460	50	Cohort	SA, SI	9
Whitmore, 2019 (78)	USA	16	50.3	12.974	2.317	Cross-sectional	SI	6
Wong, 2016 (79)	USA	15.5	NR	10.123	759	Cross-sectional	SA, SI	9

NR, Not reported; NOS, Newcastle Ottawa Scale; SA, Suicide attempt; SI, Suicidal ideation.

**Table 2:** Study characteristics of the included studies.

First author, year	Type of Sleep Disturbances	Measurement period of Sleep Disturbances	Instrument Measuring of Sleep Disturbances	N. Suicidal Behavior	Measurement period of Suicidal Behavior	Instrument Measuring of Suicidal Behavior
Altangerel, 2014 (64)	Couldn’t sleep (y/n)	Past 12 months	GSHS	Ideation (1019) Plan (659) Attempt (448)	Past 12 months	Self-structured questions
Baiden, 2020 (65)	Poor Sleep	Past 12 months	Self-structured questions	Ideation (731)	Past 12 months	Self-structured questions
Chung, 2014 (66)	Poor Sleep	In a week	Self-structured questionnaire	Ideation (108)	In a week	MINI-Kid
Dema, 2019 (80)	Insomnia	Past 12 months	Self-structured questions	Ideation (665) Attempt (654)	Past 12 months	Self-structured questions
Fitzgerald, 2011 (67)	Short total sleep times (TSTs)	Past 12 months	YRBS Self-structured questions	Ideation (2638) Attempt (1227)	Past 12 months	YRBS Self-structured questions
Franic, 2014 (68)	Sleep-related problems (DIS or DMS)	Did not specify a time frame	The Junior Eysenck Personality Questionnaire	Ideation (135)	Did not specify a time frame	Self-structured questions
Gong, 2020 (69)	Sleep disorders	Past 12 months	YRBS Self-structured questions PSQI	Ideation (66) Attempt (42)	Past 12 months	YRBS Self-structured questions
Jacob, 2020 (70)	Sleep disorders	Did not specify a time frame	Self-structured questions	Attempt (172)	2008-2017	Self-structured questions
Kim, 2015 (71)	Poor Sleep	In a week	Self-structured questions	Ideation (18408) Attempt (4169)	In a week	KCDCP KYRBWS
Kim, 2021 (72)	Sleep problem	In a week	Self-structured questions	Ideation (11650)	In a week	KYRBWS
Lin, 2018 (73)	Insomnia	Did not specify a time frame	Self-structured questions	Attempt (1395)	Did not specify a time frame	Self-structured questions
Liu, 2019 (20)	Sleep problem	Past 12 months	Self-structured questions	Attempt (347)	Past 12 months	Self-structured questions
Liu, 2021 (36)	Nightmare frequency and distress	Past 12 months	NDQ	Attempt (839)	Past 12 months	Adolescent Health Questionnaire (AHQ)
Roane, 2008 (74)	Insomnia	Past 12 months	Questions retrieved from ADD health	Ideation (569) Attempt (157)	Past 12 months	Questions retrieved from ADD health
Shi, 2021 (75)	Sleep problem	Past 12 months	PSQI	Ideation (1259) Attempt (126)	Past 12 months	Self-structured questions
Verkooijen, 2018 (76)	Sleep problem	Past 12 months	Self-structured questions	Ideation (1766) Attempt (144)	Past 12 months	Self-structured questions
Weis, 2015 (77)	Sleep problem	Past 12 months	PSQI	Attempt (50)	Past 12 months	SBQ-R
Whitmore, 2019 (78)	Poor Sleep	Past 12 months	Self-structured questions	Ideation (2317)	Past 12 months	Self-structured questions
Wong, 2016 (79)	Insomnia	Past 12 months	NCS-A	Ideation (506) Plan (101) Attempt (101)	Past 12 months	NCS-A

DIS, Difficulty initiating sleep; DMS, Difficulty maintening sleep; GSHS, Global School Health Survey; MINI-KID, Mini International Neuropsychiatric Interview-Kid; YRBS, Youth Risk Behavior Surveillance; KCDCP, Korea Centers for Disease and Control; ADD Health, Adolescent to Adult Health; KYRBWS, Korea Youth Risk Behavior Web-based Survey; NDQ, Night Distress Questionnaire; PSQI, Pittsburgh Sleep Quality Index; NCS-A, National Comorbidity Survey-Adolescent; SQB-R, Suicide Behaviors Questionnaire-Revised.

The average age of the participants was 15.2 years, and the average proportion of females was 46.3%. Of the nineteen studies examined, thirteen showed a low risk of bias based on a total score of 8-9 according to the Newcastle-Ottawa Scale (NOS). Conversely, the remaining studies exhibited a moderate risk of bias, with a total score ranging from 4 to 7. For further details, refer to the appendix, specifically **Data Sheet 1 (Supplement 1)**.

### 3.2 Association between sleep disturbances and suicidal behaviors

**Figure 2:**
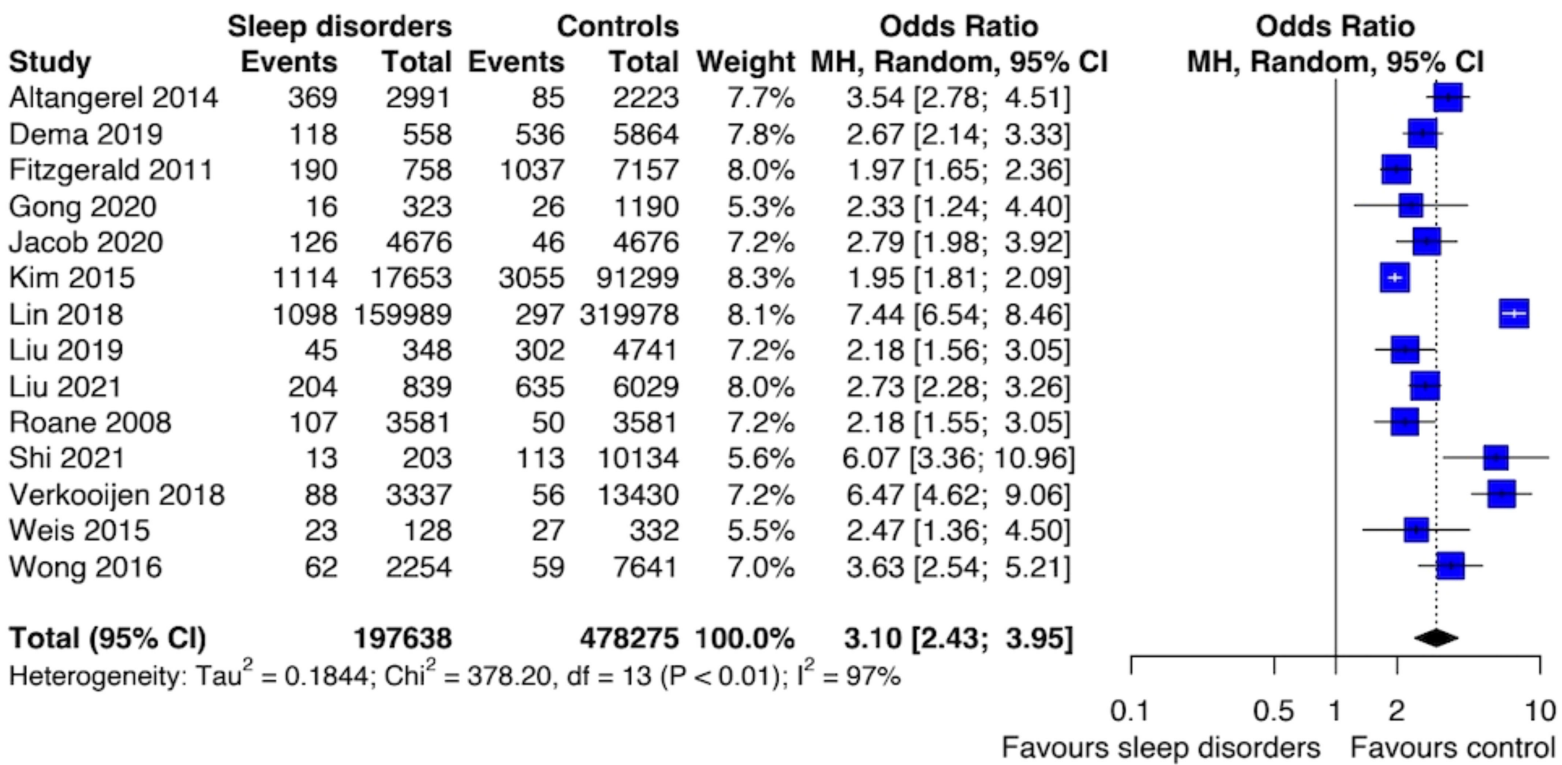
Forest plot of primary analysis between association of suicide attempt and sleep disturbances.

The prevalence of SI ranges from 5% to 19.8%, whereas the prevalence of SA ranges from 0.3% to 10.6%. The meta-analysis of the SA risk was based on 14 studies and showed an association with SDs (OR 3.10; [95% CI: 2.43; 3.95]) (**Figure 2**). The level of between-study heterogeneity was high (I^2^ = 96.6%). By looking at the forest plot of SA, it is possible to notice that the study by Lin et al. (2018) provided higher odds for SA. This study focused on adolescents with insomnia, however, the authors did not provide a timeframe to which the assessment of both SA and SDs were referred. Interestingly, this study is also the one with the largest sample size.

Sensitivity analysis removing studies with a high risk of bias confirmed the association between SDs and SA (OR: 3.03; [95% CI: 2.26; 4.06]) with a similar level of heterogeneity (I^2^ = 97%). We performed meta-regression analyses for mean age, female gender, alcohol and drug abuse, and smoking cigarettes [see **Appendix, Data Sheet 1 (Supplement 2)**].

**Figure 3:**
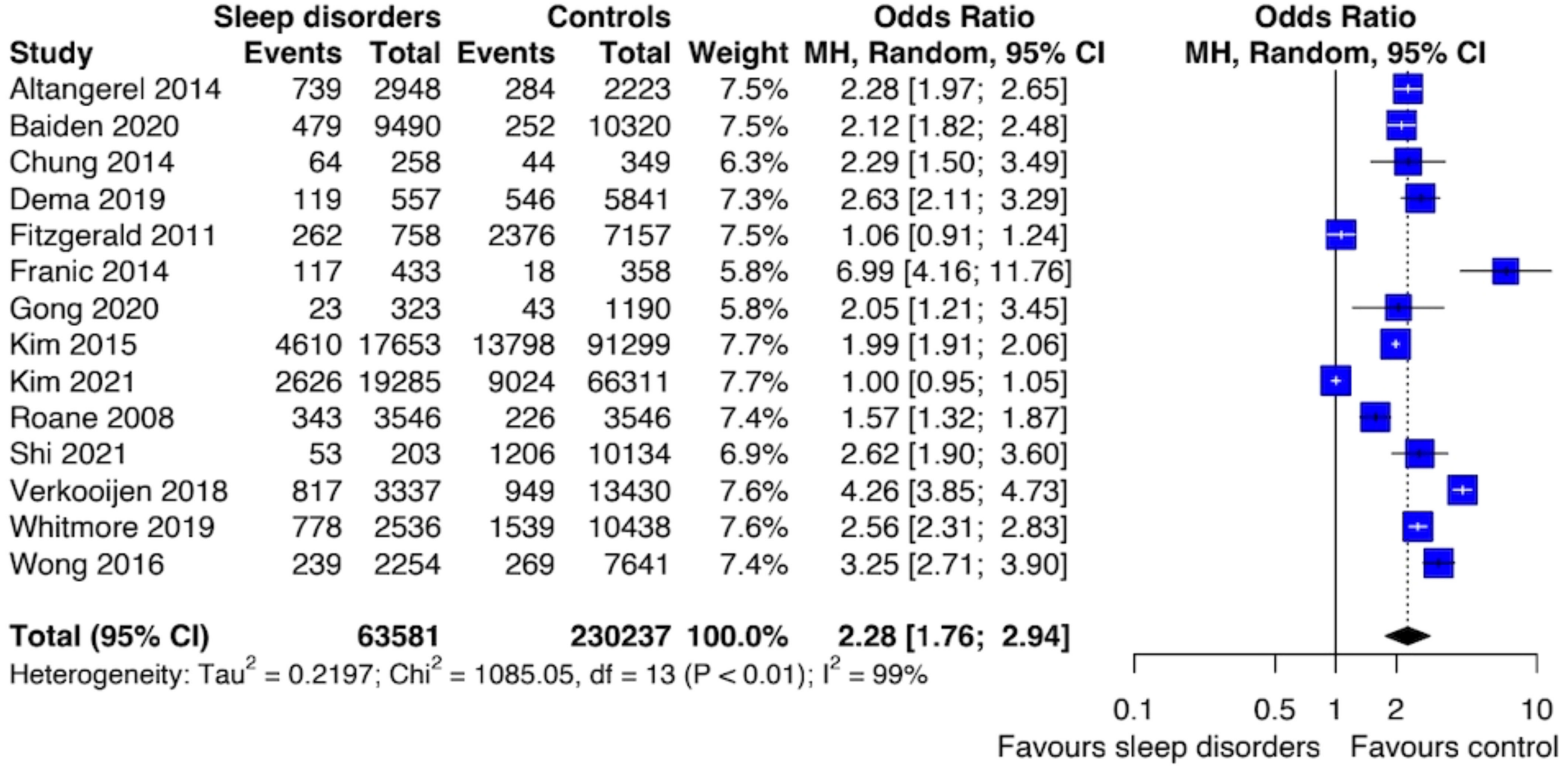
Forest plot of primary analysis between association of suicide ideation and sleep disturbances.

The results of the meta-analysis of the risk of SI were based on 14 studies and showed that individuals with SDs had an increased risk of SI compared to those unexposed to SDs (OR: 2.28; [95% CI 1.76; 2.94]) (**Figure 3**). The level of heterogeneity was high (I^2^ = 99%). Also, sensitivity analysis performed by removing studies with increased risk of bias revealed an association between SDs and SI (OR: 2.34; [95% CI: 1.69; 3.23]) with a reduction of heterogeneity (I^2^ = 94%).

There was no evidence of publication bias in either the meta-analysis, as shown by Egger’s test p-value > 0.05, and by the funnel plots displayed in **Data Sheet 1 (Supplement 3)**.

We could not perform a meta-analysis on NSSI and death by suicide as none of the included studies reported these outcomes.

## 4 Discussion

The present meta-analysis explored the association between SDs and the risk of suicidal behaviors among adolescents. Our results showed that individuals with SDs had a probability almost tripled for SA and doubled for developing SI compared to controls.

Suicidal behaviors have multifactorial causes, and our findings suggest that adequate sleep may be a protective factor that reduces suicide rates in adolescents. Mechanisms linking sleep to suicidality may differ and vary. One possibility is that being awake at night creates a window of vulnerability for suicidality. Both sleep deprivation and circadian might contribute to the hypoactivation of the frontal lobe, associated with reduced problem-solving abilities and increased impulsive behavior, possibly increasing suicide risk (45). Further, the serotoninergic system has been proposed to mediate the association between SDs and suicide (46). A previous study showed that the prefrontal cortex exhibited low serotonin synthesis in suicide attempters compared to healthy controls (47). Lower neuron density and deficient serotonin input in the prefrontal cortex, which controls executive function, may contribute to impulsive and aggressive traits that are associated with suicidal behaviors (48). Serotonin and its brain receptors also play a crucial role in sleep-wake regulation. Serotonin secretion is highest during wakefulness and decreases during sleep (49). Increasing evidence suggests that SDs result in loss of sensitivity or desensitization of postsynaptic serotonin receptors (50). Under these scenarios, SDs might lead to a loss of serotonin function and thus adversely affect impulse control, which increases the likelihood of suicide. Explanations for the relation between SDs and increased suicidality are currently speculative.

Suicidal thoughts and behaviors peak in mid-adolescence and subsequently decline in late adolescence. This observed pattern aligns with previous research findings, indicating significant developmental shifts in these phenomena during the teenage years (51). Preventive interventions for SI and SA in adolescents, particularly those without prior psychiatric treatment, are currently underdeveloped.

SDs are common in people who use specialist mental health services (52). The important role of SDs and their relation with suicidal behaviors emerges in several clinical populations, such as bipolar, schizophrenia, depressive, and anxiety disorders (53, 54). Research suggests improving sleep in people experiencing psychosis could reduce symptoms and improve functioning, however, patients frequently accept SDs as an inevitable part of their condition (55).

Given the findings of this study, the presence of SDs in adolescents may trigger the need for further evaluation of increased risk for suicide. A comprehensive suicide risk assessment may include evaluating sleep quality and maintenance. Screening measures such as the Pittsburgh Sleep Quality Index (PSQI), which assesses sleep quality, efficiency, duration, disturbances and has good reliability and validity in detecting SDs, may assist in this effort (56). A suicide risk assessment that includes an evaluation of SDs may not only add to the estimation of risk but may also provide a potential target for intervention.

Recognizing the challenges in screening and treating these behaviors in nonclinical samples, this meta-analysis emphasizes the potential role of interventions such as sleep hygiene and behavioral counseling. Sleep hygiene interventions can promote healthy sleep patterns by advocating consistent sleep schedules, caffeine avoidance before bedtime, avoidance of smartphone use in bed, and creating a conducive sleep environment (57). Furthermore, problem-solving and cognitive behavioral therapy (CBT) have shown promise in reducing repeated self-harm and suicidal ideation within a year, though engaging adolescents in these treatments can be challenging (58). Indeed, CBT for insomnia (CBT-I) has also been shown to be an effective non-pharmacological treatment for SDs (59). CBT-I typically consists of cognitive components such as cognitive restructuring, stress management, problem-solving skills, sleep education, and one or more behavioral components such as sleep restriction, relaxation training, and increasing activity levels (60). Behavioral counseling, which aims to increase homeostatic sleep drive and normal circadian rhythms, is also known to be effective in treating children and adolescents with SDs (61).

Timely intervention, before the appearance of suicide behaviors, remains a crucial consideration in mitigating the risk associated with SDs.

Our study has several limitations. Cohort and cross-sectional studies generally recruited individuals with no current suicide behaviors and collected data on SDs and suicide behaviors retrospectively through rating scales, while case-control studies recruited individuals based on current suicide behaviors and compared them with controls, collecting SDs retrospectively. Due to the cross-sectional design of most of the studies included in this meta-analysis, a causal link between SDs and suicide risk cannot be established; therefore, these results should be regarded as hypothesis-generating. Future research should prioritize longitudinal studies to elucidate the cumulative contribution of risk factors to SI and SA prediction. Otherwise, by examining data from cross-sectional research, this study emphasizes the role of SDs in leading to the risk of SA and SI. Future research should also investigate the causal association between sleep disruptions and juvenile SA, for example, using more precise measures of SDs and suicidality, prospective designs, or other methods exploiting instrumental variables, such as Mendelian randomization. Finally, future research should aim to investigate the specific impacts of various types of SDs on suicidal behaviors to provide a more nuanced understanding of these associations.

Despite the limitations highlighted, our results have relevant implications for clinical practice and policy. SDs in adolescents are a red flag that should be intercepted by pediatrists and school counters to guarantee an in-depth study of mental health for early recognition of the suicide risk associated with it. Moreover, SDs and mood changes are among the clinical criteria for early risk recognition for bipolar disorder and adolescence represents a typical moment of the first onset of the symptoms that frequently are undiagnosed (62, 63).

Preventive interventions for SI and SA in adolescents, particularly those without prior psychiatric treatment, are currently underdeveloped.

## 5 Conclusion

This study documents the role of SDs in influencing the risk of suicidal behaviors by analyzing data from many adolescents not diagnosed with psychiatric disorders.

Regarding public health implications, our findings highlight the importance of screening and managing SDs, particularly insomnia, warranting future research on the impact of that on suicide prevention. Additional prospective studies are required to establish the causal relationship between SDs and youth suicide plans and attempts using reliable SDs and suicidality measures.

## Data Availability

The original contributions presented in the study are included in the article/**Supplementary Material**. Further inquiries can be directed to the corresponding author.
